# Healthy Food on the Twitter Social Network: Vegan, Homemade, and Organic Food

**DOI:** 10.3390/ijerph18073815

**Published:** 2021-04-06

**Authors:** Ladislav Pilař, Lucie Kvasničková Stanislavská, Roman Kvasnička

**Affiliations:** 1 Department of Management, Faculty of Economics and Management, Czech University of Life Sciences Prague, 165 21 Prague, Czech Republic; kvasnickova@pef.czu.cz; 2 Department of Systems Engineering, Faculty of Economics and Management, Czech University of Life Sciences Prague, 165 21 Prague, Czech Republic; kvasnicka@pef.czu.cz

**Keywords:** healthy food, vegan, organic food, homemade food, social media analysis, Twitter

## Abstract

Online social networks have become an everyday aspect of many people’s lives. Users spend more and more time on these platforms and, through their interactions on social media platforms, they create active and passive digital footprints. These data have a strong potential in many research areas; indeed, understanding people’s communication on social media is essential for understanding their attitudes, experiences, behaviors and values. Researchers have found that the use of social networking sites impacts eating behavior; thus, analyzing social network data is important for understanding the meaning behind expressions used in the context of healthy food. This study performed a communication analysis of data from the social network Twitter, which included 666,178 messages posted by 168,134 individual users. These data comprised all tweets that used the #healthyfood hashtag between 2019 and 2020 on Twitter. The results revealed that users most commonly associate healthy food with a healthy lifestyle, diet, and fitness. Foods associated with this hashtag were vegan, homemade, and organic. Given that people change their behavior according to other people’s behavior on social networks, these data could be used to identify current and future associations with current and future perceptions of healthy food characteristics.

## 1. Introduction

Food is a part and necessity of every person’s daily life [1]. Therefore, from a health and policy point of view, it is necessary to investigate what people eat and their opinions about individual foods. Unhealthy eating is a problem in many countries [2,3], and if national governments need to develop action programs to promote healthy eating, a thorough understanding of consumer attitudes, experiences, and behaviors regarding healthy foods is needed. One way to obtain such information is through social media analysis [4]. Social media is now a part of many individuals’ everyday lives. Increasingly, users spend more time on these platforms, and create active and passive digital footprints through their interactions with other platform users [5]. These data have a strong research potential in many areas, especially considering that understanding people’s communication on social media is essential for understanding their attitudes, experiences, behaviors, and values [6,7]. In the field of food, studies have already analyzed social network data in the context of farmers’ markets [8], organic food [9], undergraduate students’ food choices [10], food sharing [11], and food security [12]. At present, social networks are used by around 3.6 billion people worldwide, and it has been predicted that more than 4.41 billion people will use social networks in 2025 [13]. When comparing this predicted number of social network users with the predicted global population for 2025 (8,184,437,460 inhabitants) [14], this indicates that approximately 54% of the planet’s population will use social networks by 2025. This is therefore a pertinent source of data with the potential to increase both the number of users and shared content.

The aims of this study as to identify the main topic associated with healthy food on the Twitter social network.

### 1.1. Theoretical Background

The increase in the number of people with obesity is a global trend [15,16,17]. Many studies have underlined the association between obesity and health problems, such as cardiovascular disease [18,19], metabolic diseases [20,21], cancer [22,23], and a higher risk of severe COVID-19 [24,25,26,27]. Although obesity has been considered as primarily a problem in Western countries, urbanization is setting the stage for an obesity epidemic in Asia [28,29,30], in some African countries such as Sudan [31], and in India [32], and is thus becoming a global problem [16,33]. Food environments and dietary patterns are closely linked to obesity [34], and food is also reflected in our way of life, culture, and well-being. For these reasons, there is now a significant interest in public health [1]. Healthy foods are an important determinant of good health [35,36], while unhealthy eating can lead to significant health risks [37,38,39]. Understanding the factors that influence the food choices and eating behaviors of consumers is essential for tackling this issue [40]. Previous studies have revealed that customers’ food selection process is greatly influenced by their social environment, including friends and the shopping environment (such as in-store marketing activities) [41,42,43,44,45]. Furthermore, two key studies reported that social networks have an important impact on consumers’ shopping behavior [45,46]. Social networks have now become environments in which users can independently create and share content. By 2025, a predicted 4.41 billion people will use social networks [13], which is half the global population. Since young adults have widely adopted social media, health researchers are looking for ways to leverage this engagement with social media to deliver interventions and health promotion campaigns [46]. The global problem with obesity and healthy problems are so central reasons why many studies focus on the use of social networks and health issues [47]. because, social media has the potential to exchange information related to healthy behaviors [48,49]. Based on the above analysis of communication on social networks about the subject of food could help us to gain a deeper understanding of consumer behaviors and attitudes [50] when it comes to healthy food.

#### 1.1.1. Social Media and Food Behavior

Social media can influence consumer behavior, such as choosing food and shopping behavior, which has been shown in following studies. Fleming-Milici and Harris [51] identified adolescents’ social media engagement with food/beverage brands. Simeon and Scarpato [52] reported the negative effects of gathering information on social networks, which tend to homogenize consumption and decrease consumers’ sustainability awareness. Social networks can be searched for recipes [53] and information on diets [54], and can be a good source of dietary information, such as food reviews [52] or advice for feeding young children [55].

Thus, social media can influence what food consumers buy and eat [53,56], and can be used as a policy measure to improve food literacy [57,58], and encourage healthy eating and a healthier lifestyle in general [10]. Indeed, some studies have investigated how social media can be used to promote health issues, such as nutrition interventions for adolescents and young adults [59] or health issues during the Covid-19 pandemic [60].

#### 1.1.2. Social Media Analysis

Social networks and social media are highly interrelated concepts. An example is the Twitter platform, which is a social medium that is built on users’ social networks. On the Twitter platform, users create messages called “tweets”. The tweet can have a maximum of 280 characters (including hashtags). In order for Twitter to function as a social medium, where content will be shared by millions of users, it is first necessary to create this network of users through their mutual relations, which is the essence of the social network. Two basic areas of research are used in area of social media, as follows: (1) social network analysis is the process of examining social structures using network theory and graph theory [61]. Social network analysis represents a network of people as graphs and examines their connections. The nodes in this network represent people, and the edges connecting the two nodes represent the relationships between them [62]—for example, who has the most friends, who people follow the most or segmentation of users, on the basis of their political affiliation [63,64]; and (2) social media analysis, the goal of social media analysis is to monitor, analyze, and represent data from social media that offer useful information on patterns of behavior [65]. For example extracting human feelings through the unstructured text—sentiment analysis [66], or analyzing what people write in relation to a particular event (whether tweets contain information, opinion, or action-related content about the event) [67]. When analyzing data from social media, we can focus on analyzing the structure of users of a platform using social network analysis (e.g., how highly a person is connected within a network), or on analyzing the content of the communication using social media analysis (e.g., user opinions and moods).

Social media analysis thus helps us to gain deeper insights into social, cultural, and environmental issues [50]. Data from the social network Twitter have shown to be a suitable source of knowledge about food-related consumer behaviors [68,69,70]. These data can provide scientists with a vast amount of information on individuals’ opinions, moods, activities [71], and experiences [72].

## 2. Materials and Methods

**Figure 1:**
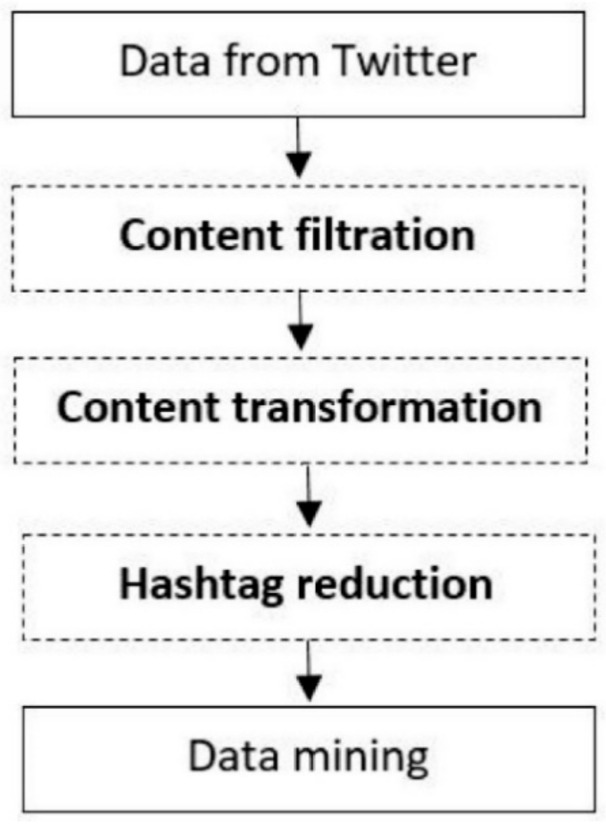
The data analysis process.

**Figure 2:**
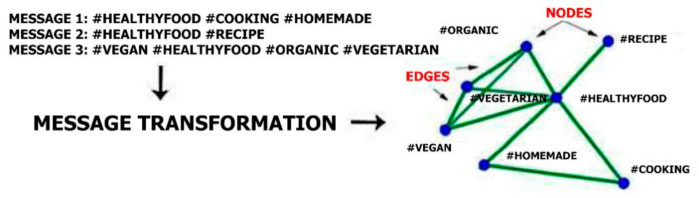
Transformation from the Twitter social network into a hashtag network.

The data analysis was based on the Knowledge Discovery in Databases process [73], and was modified to the requirements of social media data analysis with a focus on hashtags (see Figure 1). The hashtag is a specific part of the message text that begins with a “#” character. In social media, the hashtag has two primary functions, firstly to filter posts, where the algorithms of social networks display an archive of messages related to this hashtag (topic) based on a specific hashtag [74]. The second function of hashtags is the possibility to express experience, attitudes, opinions, and values via social media [6,7,75,76], in areas that the user wants to emphasize on social networks. For example, emphasize that the food I put on social media is vegan through the hashtag #vegan. This procedure has already been used in research focusing on farmers’ markets [8], organic foods [9], sustainability [77], gamification [78], and corporate social responsibility [79]. The data analysis process consisted of five steps, as follows:

1. The Twitter API [80] was used to obtain messages (Tweets) from communications on the social network Twitter. The data were recorded between 1 January 2019 and 31 December 2020. The software captured messages that used the hashtag #healthyfood. During that period, 666,178 Tweets of 168,134 unique users were captured. This dataset contains all messages that contained the hashtag #healhtyfood in the monitored period, which users sent to the Twitter social network.
2. Content filtration: As our analysis only focused on hashtags, all words that were not preceded by the hashtag symbol (“#”) were removed. This led to a dataset that consisted purely of hashtags (i.e., words beginning with the symbol #).
3. Content transformation: Subsequently, all letters were transformed into lower-case letters to prevent potential duplicates (e.g., the software might consider #Vegan, #vegan, and #VEGAN as three different hashtags). A further correction was made to break up strings of connected hashtags, e.g., “#vegan#organic” was converted to “#vegan; #organic”. The dataset was imported into Gephi 0.9.2, where a hashtag network was created based on hashtag interdependence (see Figure 2). Gephi is an open-source leading visualization and exploration software for graphs and networks [81]. To use social network analysis methods, it was necessary to create a network of hashtags based on the rule: Nodes = Hashtags and Edges = their representation in one report. For example—Message: “I love homemade cooking #healthyfood #homemade #cooking” 3 Nodes (#healthyfood, #homemade and #cooking) are inserted into the graph and edges are created between these hashtags (because they are all in one message)—see Figure 2. If the following message contains the text: “These cookies are wonderful, if you want, I can share my #healthyfood #recipe to you”, the hashtag #recipe will be inserted into the graph, which will be connected only with the hashtag #healthyfood, which already in the chart exists from the last report. For the #healthyfood hashtag, the frequency value changes from 1 to 2.

1. Hashtag reduction: To detect communities is necessary to process a hashtag reduction that removes micro-communities. A large number of micro-communities is caused by an extensive number of hashtags that contain local hashtags, for example, farmers’ markets in the Czech Republic—Prague Dejvice—#farmersmarketpraguedejvice #applefromfarmersmarketpraguedejvice or hashtags created by the users themselves #nameandsurname.
2. Data mining: The following methods were used to describe the network:
  (a) Frequency: The frequency is a value that expresses the hashtag frequency within a network.
  (b) Eigenvector centrality: This is an extension of degree centrality, which measures the influence of hashtags in a network. The value is calculated based on the premise that connections to hashtags with high values (hashtags with a high degree of centrality) have a greater influence than links with hashtags of similar or lower values. A high eigenvector centrality score means that a hashtag is connected to many hashtags with a high value, and was calculated as follows:
    (1) $$x_{v}=\frac{1}{\lambda }\underset{t\in M\left(v\right)}{\sum }x_{t}=\frac{1}{\lambda }\underset{t\in G}{\sum }a_{v,t}x_{t}$$
    where *M*(*v*) denotes a set of adjacent nodes, and *λ* is a largest eigen value. Eigenvector *x* can be expressed by Equation (2), as follows:
    (2) Ax=λx
    This value was used to identify important hashtags for community analysis (see next step).
  (c) Modularity and community analysis: The most complex networks contain nodes that are mutually interconnected to a larger extent than they are connected to the rest of the network. Groups of such nodes are called communities [82]. Modularity represents an index that identifies the cohesion of communities within a given network [83]. The idea is to identify node communities that are mutually interconnected to a greater degree than other nodes. Networks with high modularity show strong links between nodes inside modules but weaker links between nodes in different modules [84]. The component analysis then identifies the number of different components (in the case of community modularity) in the network based on the modularity detection analysis [85], as follows:
    (3) $$\Delta Q=\left[\frac{\sum_{in}+2k_{i,in}}{2m}-{\left(\frac{\sum_{tot}+k_{i}}{2m}\right)}^{2}\right]-\left[\frac{\;\sum_{in}}{2m}-{\left(\frac{\sum_{tot}}{2m}\right)}^{2}\right]$$
    where ∑*in* is the sum of weighted links inside the community, *∑tot* is the sum of weighted links incident to hashtags in a community, *k_i_* is the sum of weighted links incident to hashtag i, *k_i,in_* is the sum of weighted links going from i to hashtags in a community, and *m* normalizing factor is the sum of weighted links for the whole graph.
  (d) Visualization of the network: The goal of network visualization is to identify individual communities and their mutually position. After importing the data into the Gephi program, the visualization of the network is concentrated in the basic square without visualizing the different relationships between individual hashtags. This visualization is unsatisfactory in terms of identifying communities and their mutual positions, but does not affect the analysis of hashtag-level and network-wide characteristics. In the field of visualization, it is possible to use the ForceAtlas2 algorithm. ForceAtlas2 is an improved version of the ForceAtlas algorithm, which focuses on large networks. This method is based on the visual representation of reduced samples to define network communities and their types [86]. The advantage over ForceAtlas is its speed and ease of computing. The ideal number of hashtags is 10,000–100,000 [26].

## 3. Results and Discussion

First, a basic analysis was performed based on the frequency of occurrence of individual hashtags in 2019 and 2020 (Table 1).

The analysis of the top 15 hashtags revealed that the frequency of the use of all hashtags except #recipe increased from 2019 to 2020 (see Table 1).

A basic analysis of the frequency of the use of individual hashtags in 2020 revealed that the hashtag #healthylifestyle was the most used hashtag in connection with the hashtag #healthyfood. According to Bektas et al. [87], healthy eating is one of the dimensions of a healthy lifestyle, as is nutrition and physical activity. This hashtag (#nutrition) was also very often used in conjunction with the hashtag #healthyfood in 2020 and was thus the 5th most used hashtag. A weight loss area has also been identified in connection with healthy food—the #weightloss hashtag (8th place) and #diet (6th place). This area may be associated with obesity. The study by Karami et al. (2018) [88] focused on analyzing the characteristics of the general public’s opinions regarding understanding public health opinions on social media as expressed on Twitter, which showed a notable correlation between diet and obesity was determined. As we mentioned in the introduction, obesity is a worldwide problem [15,16,17], and there is an association between obesity and health problems [21,22,27]. Weight loss is considered to be the cornerstone of treatment for obese people [89,90]. Here it is important to draw attention to the perception of oneself on social networks [91]. Thus, weight loss may not be associated with obesity but may be connected with the need to get a figure in the underweight (by BMI) area [92]. For this reason, it is advisable to analyze the area separately with a focus on the #weightloss area. Other important hashtags used in connection with #healthyfood were #vegan (4th place) and #vegetarian (15th place). This connection confirms many previous studies that have identified health-related reasons as one of the main motivations for following both a vegan diet [93,94,95] and a vegetarian diet [96,97]. Other categories of hashtags that were often used in connection with the #healthyfood hashtag in 2020 were #recipe (7th place), #cooking (9th place), and #homemade (10th place). The high frequency of these hashtags may be related to the lifestyle changes caused by governmentally mandated lockdowns in 2020 that led to people being forced to cook a significant proportion of their food at home [98,99]. Many parents had to provide home-based meals for their children and, according to a study by Phlippe et al. [100], many parents observed changes in diet; for example, boredom led to increased consumption and emotional overeating. Laguna et al. [98] also identified changes in food purchases during the 2020 lockdown, whereby consumers bought more fruits and vegetables and fewer pastries and desserts for health reasons. This can be related to the frequent use of the hashtag #recipe (8th place), as people often search for and share recipes on the social networks [53]. The most common hashtags used in connection with the hashtag #healthyfood were #vegan (4th place), #homemade (10th place), and #organic (12th place).

### 3.1. Community Analysis

The community analysis extrapolated the following five communities: (1) Active lifestyle, (2) Influencer, (3) Diets, (4) Recipes, and (5) Health problems (Table 2).

**Figure 3:**
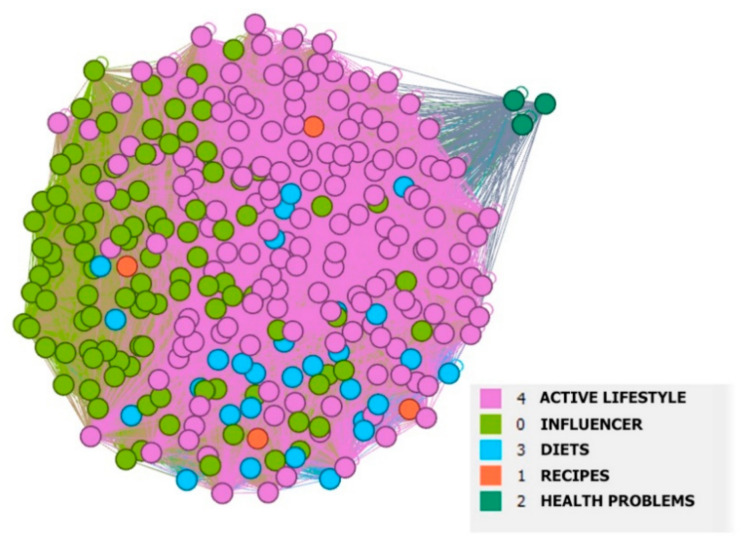
Community visualization on the Twitter social network in the area of healthy food.

The largest community was that focused on an “Active lifestyle”. This community contained hashtags that were associated with areas such as healthy lifestyle, fitness, healthy living, nutrition, healthy eating, wellness, and lifestyle. The second-largest community was the “Influencer” community, in which people shared foods they considered healthy. This community contained hashtags such as #foodporn, #delicious, #yummy, and #foodlover. Hashtags focused on homemade, cooking, and food blogger are likely to be related to food cooked and shared with other people via Twitter, and these users can be considered as food bloggers (influencers). Influencer marketing is built on building trust between the influencer and the follower, to whom the influencer then passes on their knowledge, experience, attitudes, opinions, and values via social media [4,7,75,76]. The smaller (16.74%) third community was focused on “Diets”, which included areas such as vegan, organic, vegetarian, plant-based, gluten-free, sugar-free, wholefood, and clean eating. The field of organic food was separately analyzed in the previous research [9], which identified a high connection in the communication of organic food with healthy food and vegan food. For the whole diet community, it is necessary to draw attention to its high connection in the active lifestyle, see Figure 3, which again leads to the question of the extent to which people consider vegan a diet in terms of the absence of certain foods and to what extent vegan in terms of lifestyle. The last, very small community (2.61%) is the community focused on “Health problems”, which included areas such as high blood pressure, blood pressure, hypertension, and diabetes. As already mentioned, healthy food affects health [35], including the issues mentioned in this community, such as diabetes [101] and hypertension [102]. The frequent use of social media to address health problems has also been confirmed by Korda and Itani [103].

#### Visual Analysis

Visualization of the network (Figure 3) was performed to gain an insight into the polarity of individual communities. The value of modularity was low (0.239), which means that hashtags were interconnected both within communities and between communities. As seen in Figure 2, the “Diets” community was more closely linked to the “Active lifestyle” community than to the “Influencer” community. The community with a focus on “Recipes” was relatively evenly distributed among all communities, except for the “Health problems” community, which was relatively isolated from the other communities.

In terms of food characteristics, the most commonly used hashtag in connection with the hashtag #healthyfood was #vegan, which refers to the vegan diet. Vegans eat no animal products, while vegetarians do not eat animals [104]. As mentioned in the theoretical background, previous studies have shown that vegan and vegetarian diets are expanding societal phenomena, particularly in Western developed countries [105,106]. Previous work has shown that the number of consumers who lean toward vegan and vegetarian diets continues to grow [107]; moreover, veganism has exhibited a greater increase in a shorter time period [108]. According to one study [109], veganism is not only a diet, but also a lifestyle; veganism can be considered as a broader involvement in cognitive and behavioral aspects of social worlds. Following this, further studies should also directly focus an analysis on the area of #healthylifestyle and #healthyliving. A comparison of the results from healthy living and healthy lifestyle area would provide important information about the relationship between healthy food and lifestyle. When comparing the time evolution of hashtags in both studies, it could be defined by how lifestyle affects healthy eating.

It is thus a matter of opinion as to whether veganism is considered as a “diet”, which is the position of Lemale [110] and Lederer [111], or as a lifestyle, as it has been described by Costa [109].

### 3.2. Study Limitations

Finally, some limitations of our study deserve attention. First of all, like other social media analyses, this study focuses only on one social network [8,9,68,77,78,112] and on English keywords. Twitter is specific to a maximum message limit of 280 characters and also focuses on text. Instagram, on the other hand, is limited to 2200 characters, which can provide other information.

Second, this study did not deal with the geolocation of sending messages. In the following studies, it would be appropriate to focus on the use of geolocation in the report to obtain other important conclusions. This limitation results in the impossibility of verifying the proportionality of the representation of individual regions in the data set.

Third, the study analyzes the current situation and does not deal with predictions for the future. In our future research, we will focus on using artificial intelligence to predict trends in social network communication.

### 3.3. Future Research

The results of this study opened many important questions for further research through both quantitative and qualitative methods.

The present research identified main values/characteristics, that Twitter users express in the healthy food area through hashtags. In future research, it will be necessary to focus on these identified areas: healthy lifestyle, vegan, vegetarian, plant-based, gluten-free, sugar-free, wholefood, and clean eating. Since the healthy food issue is a very complex area, it will be appropriate to focus research on both the analysis of individual areas separately and the analysis of their interconnectedness. To broaden our understanding of the healthy food area, it is also necessary for researchers to focus on other social networks, such as Instagram, TikTok, Yandex, and Facebook. These results may be important for understanding the common areas that connect the individual characteristics. These interfaces are important for media communication and understanding of the common values of social network users in the monitored area.

Like other studies [7,8,64,70,71,102], these results are analyzed from a global perspective. In the following research, it would be useful to find out whether regional differences in food perception can be found, or whether differences can be found, for example, between Europe and the US, or whether it is possible to create new regional segmentation, based segmentation of users with the reverse assignment of location. In other words, as Khokhar and Serajuddin (2015) [113] points out that categorization by developed and developing countries, for example, is obsolete. Subsequent research could create a map of perceptions of individual food characteristics across regions.

Another important area identified by this study is the connection between healthy food and healthy lifestyle (the #healthylifestyle hashtag is the most widely used hashtag in conjunction with #healthyfood). Based on this, further studies can focus on vegan, vegetarian, or gluten-free areas and focus on understanding whether these areas are considered by people to be “more healthy food”, such as [9], or it is a way of life that is not only based on one’s own health, but also on the protection of nature and animals, where people avoid the use of animals in any context, including food, clothing, sport, and entertainment [114]. This understanding is important both in terms of marketing communication of product manufacturers and in understanding these trends on social networks in the field of healthy food.

A weight loss area has also been identified in connection with healthy food, which may or may not be associated with obesity. Here it is important to draw attention to the perception of oneself on social networks [115]. Thus, weight loss may not be associated with obesity but may be connected with the need to get a figure in the underweight (by BMI) area [91]. For this reason, it is advisable to analyze the area separately with a focus on the #weightloss area.

## 4. Conclusions

Consumer behavior is a dynamic system, whereby consumers interact with producers, farmers, traders, retailers, industries, governments, and a series of other actors [92], and must, therefore, be monitored in terms of understanding people’s perceptions and identifying the current situation. Social media, which is used by more people every year, has substantial research potential for basic quantitative analyses, which can be followed up by qualitative research. The present study identified new research questions and fundamental information that could be used as a basis for the following analysis in the field of Public Health and Policy.

We found that the #healthylifestyle and #healthyliving hashtags were the most commonly used hashtags in conjunction with the #healthyfood hashtag. This is an important finding that identifies that people on social networks connect food with lifestyle, which is not just about nutrition, but about the way a person lives.

The “Influencer” community, with a focus on influencer marketing, was the second-largest community in the present study. Influencer marketing has a large impact on customer behavior [116]. Influencer marketing is built on building trust between the influencer and the follower, to whom the influencer passes on their knowledge, experience, attitudes, opinions, and values via social media [4,7,75,76]. As this was identified as the second-largest community, people interested in the area of healthy food may be strongly influenced by influencers.

Based on the analysis of the social network Twitter, with a focus on healthy food through the hashtag #healthyfood, the three basic characteristics of food that were most communicated in 2020 were vegan, homemade, and organic. This is an important finding because it indicates how social network users perceive healthy food. These results confirm previous research [117,118,119] that has reported that people perceive vegan food, organic food, and homemade food as the healthiest types of food. Another important finding is the identification of a t difference in the use of the vegan hashtag (4th place), which expresses the rejection of the use of animal products, and vegetarian (15th place), which expresses only the rejection of eating animals [104].

Another characteristic associated with healthy food was organic. This is a continuously growing trend, which confirms previous studies [120,121]. According to an analysis of the social network Instagram [9], #organicfood is most often associated with the hashtag #organic and, subsequently, with the hashtag #healthyfood. Organic is an important characteristic of food that users communicate on social networks.

To summarize, we found that users most commonly associate healthy food with a healthy lifestyle, fitness, nutrition and diet. Foods associated with this hashtag were vegan, homemade, and organic. The community analysis extrapolated the following five communities: (1) Active lifestyle, (2) Influencer, (3) Diets, (4) Recipes, and (5) Health problems. Based on these results, it is possible to identify lifestyle as an important element in the field of healthy eating. In the following studies, it is necessary to focus separately on individual topics that have been identified as the most commonly associated with healthy food and to make a comparison within other social networks like Facebook, Instagram, and TikTok.

## Figures and Tables

**Table 1:** Hashtags published in connection with the hashtag #healthyfood in 2019 and 2020.

	2019		2020		
	Abs	Rel	Abs	Rel	Difference
Healthylifestyle *	42,471	0.143	64,990	0.253	0.11
healthyliving	20,196	0.068	31,853	0.124	0.056
fitness	30,591	0.103	31,082	0.121	0.018
vegan	27,918	0.094	26,458	0.103	0.009
nutrition	22,572	0.076	25,431	0.099	0.023
diet	20,196	0.068	22,862	0.089	0.021
weightloss	15,444	0.052	20,293	0.079	0.027
recipe	25,839	0.087	18,495	0.072	−0.015
cooking	17,523	0.059	16,954	0.066	0.007
homemade	11,880	0.04	13,615	0.053	0.013
wellness	10,098	0.034	12,587	0.049	0.015
organic	11,880	0.04	12,073	0.047	0.007
motivation	8316	0.028	12,073	0.047	0.019
healthcare	9207	0.031	10,789	0.042	0.011
vegetarian	10,989	0.037	10,275	0.04	0.003

* Sorted by Abs values—2020.

**Table 2:** Communities extracted from the reduced network.

Number of Communities *	Size of Community	Name of Community	Key Hashtags
4	39.60%	Active lifestyle	#healthylifestyle, #fitness, #healthyliving, #nutrition, #weightloss, #healthyeating, #wellness, #lifestyle
0	34.81%	Influencer	#foodporn, #delicious, #yummy, #foodblogger, #homemade, #foodlover, #cooking
3	16.74%	Diets	#vegan, #organic, #vegetarian, #plantbased, #glutenfree, #sugarfree, #wholefood, #cleaneating
1	6.24%	Recipes	#recipe, #recipes, #recipeoftheday, #recipeoftheweek
2	2.61%	Health problems	#highbloodpressure, #bloodpressure, #hypertension, #diabetes

* Linked to Figure 3.

## Data Availability

All data used in this study can be downloaded via the Twitter API [80].

## References

[1] Abbar S., Mejova Y., Weber I. You Tweet What You Eat. Proceedings of the 33rd Annual ACM Conference on Human Factors in Computing Systems.

[2] Strickland J.R., Eyler A.A., Purnell J.Q., Kinghorn A.M., Herrick C., Evanoff B.A. (2015). Enhancing Workplace Wellness Efforts to Reduce Obesity: A Qualitative Study of Low-Wage Workers in St Louis, Missouri, 2013–2014. Prev. Chronic Dis..

[3] Crovetto M., Valladares M., Espinoza V., Mena F., Oñate G., Fernandez M., Durán-Agüero S. (2018). Effect of healthy and unhealthy habits on obesity: A multicentric study. Nutrition.

[4] Maynard D., Roberts I., Greenwood M.A., Rout D., Bontcheva K. (2017). A framework for real-time semantic social media analysis. J. Web Semant..

[5] Apuke O.D. (2017). Social and Traditional Mainstream Media of Communication: Synergy and Variance Perspective. Online J. Commun. Media Technol..

[6] Zhang K., Geng Y., Zhao J., Liu J., Li W. (2020). Sentiment Analysis of Social Media via Multimodal Feature Fusion. Symmetry.

[7] Pilař L., Poláková J., Gresham G., Rojík S., Tichá I. (2017). Why People Use Hashtags When Visiting Farmers’ Markets. Agrarian Perspectives XXVI: Competitiveness of European Agriculture and Food Sectors.

[8] Pilař L., Balcarová T., Rojík S., Tichá I., Poláková J. (2018). Customer experience with farmers’ markets: What hashtags can reveal. Int. Food Agribus. Manag. Rev..

[9] Pilař L.K.S.L., Rojík R.K.S., Gresham G. (2018). Customer Experience with Organic Food: Global View. Emir. J. Food Agric..

[10] Blundell K.-L., Forwood S. (2021). Using a social media app, Instagram, to affect what undergraduate university students choose to eat. Appetite.

[11] Harvey J., Smith A., Goulding J., Illodo I.B. (2020). Food sharing, redistribution, and waste reduction via mobile applications: A social network analysis. Ind. Mark. Manag..

[12] Jiren T.S., Bergsten A., Dorresteijn I., Collier N.F., Leventon J., Fischer J. (2018). Integrating food security and biodiversity governance: A multi-level social network analysis in Ethiopia. Land Use Policy.

[13] Tankovska H. Number of Global Social Network Users 2017–2025. https://www.statista.com/statistics/278414/number-of-worldwide-social-network-users/.

[14] Current World Population Population. https://www.worldometers.info/world-population/.

[15] Finucane M.M., Stevens G.A., Cowan M.J., Danaei G., Lin J.K., Paciorek C.J., Singh G.M., Gutierrez H.R., Lu Y., Bahalim A.N. (2011). National, regional, and global trends in body-mass index since 1980: Systematic analysis of health examination surveys and epidemiological studies with 960 country-years and 9.1 million participants. Lancet.

[16] Wang Y.C., McPherson K., Marsh T., Gortmaker S.L., Brown M. (2011). Health and economic burden of the projected obesity trends in the USA and the UK. Lancet.

[17] Revels S., Kumar S.A., Ben-Assuli O. (2017). Predicting obesity rate and obesity-related healthcare costs using data analytics. Health Policy Technol..

[18] Lee H., Choi E.-K., Lee S.-H., Han K.-D., Rhee T.-M., Park C.-S., Lee S.-R., Choe W.-S., Lim W.-H., Kang S.-H. (2017). Atrial fibrillation risk in metabolically healthy obesity: A nationwide population-based study. Int. J. Cardiol..

[19] Mirzaei B., Abdi H., Serahati S., Barzin M., Niroomand M., Azizi F., Hosseinpanah F. (2017). Cardiovascular risk in different obesity phenotypes over a decade follow-up: Tehran Lipid and Glucose Study. Atherosclerosis.

[20] Frank A.P., Santos R.D.S., Palmer B.F., Clegg D.J. (2019). Determinants of body fat distribution in humans may provide insight about obesity-related health risks. J. Lipid Res..

[21] Cho W.K., Han K., Ahn M.B., Park Y.-M., Jung M.H., Suh B.-K. (2018). Metabolic risk factors in Korean adolescents with severe obesity: Results from the Korea National Health and Nutrition Examination Surveys (K-NHANES) 2007–2014. Diabetes Res. Clin. Pr..

[22] Ezzati M., Lopez A.D., Rodgers A., Hoorn S.V., Murray C.J. (2002). Selected major risk factors and global and regional burden of disease. Lancet.

[23] Formica V., Morelli C., Riondino S., Renzi N., Nitti D., Di Daniele N., Roselli M., Tesauro M. (2020). Obesity and common pathways of cancer and cardiovascular disease. Endocr. Metab. Sci..

[24] Le Brocq S., Clare K., Bryant M., Roberts K., Tahrani A. (2020). Obesity and COVID-19: A call for action from people living with obesity. Lancet Diabetes Endocrinol..

[25] Yu W., Rohli K.E., Yang S., Jia P. (2021). Impact of obesity on COVID-19 patients. J. Diabetes Complicat..

[26] Jacomy M., Venturini T., Heymann S., Bastian M. (2014). ForceAtlas2, a Continuous Graph Layout Algorithm for Handy Network Visualization Designed for the Gephi Software. PLoS ONE.

[27] Mulugeta W.M. (2020). Obesity Management in Primary Care During and Beyond the COVID-19 Pandemic. J. Nurse Pr..

[28] Fan J.-G., Kim S.-U., Wong V.W.-S. (2017). New trends on obesity and NAFLD in Asia. J. Hepatol..

[29] Tee E.-S. (2002). Obesity in Asia: Prevalence and issues in assessment methodologies. Asia Pac. J. Clin. Nutr..

[30] Wu Y., Wang L., Zhu J., Gao L., Wang Y. (2021). Growing fast food consumption and obesity in Asia: Challenges and implications. Soc. Sci. Med..

[31] Ali Y.A., Almobarak A.O., Awadalla H., Elmadhoun W.M., Ahmed M.H. (2017). Obesity among Sudanese adults with diabetes: A population-based survey. Ann. Transl. Med..

[32] Peltzer K., Pengpid S., Samuels T.A., Özcan N.K., Mantilla C., Rahamefy O.H., Wong M.L., Gasparishvili A. (2014). Prevalence of Overweight/Obesity and Its Associated Factors among University Students from 22 Countries. Int. J. Environ. Res. Public Health.

[33] Gormley N., Melby V. (2020). Nursing students’ attitudes towards obese people, knowledge of obesity risk, and self-disclosure of own health behaviours: An exploratory survey. Nurse Educ. Today.

[34] Jessri M., Wolfinger R.D., Lou W.Y., L’Abbé M.R. (2017). Identification of dietary patterns associated with obesity in a nationally representative survey of Canadian adults: Application of a priori, hybrid, and simplified dietary pattern techniques. Am. J. Clin. Nutr..

[35] Wyckhuys K.A., Aebi A., van Lexmond M.F.B., Bojaca C.R., Bonmatin J.-M., Furlan L., Guerrero J.A., Mai T.V., Pham H.V., Sanchez-Bayo F. (2020). Resolving the twin human and environmental health hazards of a plant-based diet. Environ. Int..

[36] De L.C., De T. (2019). Healthy Food for Healthy Life. J. Glob. Biosci..

[37] Cecchini M., Sassi F., Lauer J.A., Lee Y.Y., Guajardo-Barron V., Chisholm D. (2010). Tackling of unhealthy diets, physical inactivity, and obesity: Health effects and cost-effectiveness. Lancet.

[38] Gupta A., Braunack-Mayer A., Smithers L., Harford J., Coveney J. (2020). Good and bad sugars: Australian adults’ perspectives on sugar in their diet. Crit. Public Health.

[39] Roberto C.A. (2020). How psychological insights can inform food policies to address unhealthy eating habits. Am. Psychol..

[40] Carrillo E., Varela P.F., Salvador A., Fiszman S.M. (2011). Main Factors Underlying Consumers’ Food Choice: A First Step For The Understanding Of Attitudes Toward “Healthy Eating”. J. Sens. Stud..

[41] Gonçalves D., Coelho P., Martinez L.F., Monteiro P. (2021). Nudging Consumers Toward Healthier Food Choices: A Field Study on the Effect of Social Norms. Sustainability.

[42] Wongprawmas R., Mora C., Pellegrini N., Guiné R.P.F., Carini E., Sogari G., Vittadini E. (2021). Food Choice Determinants and Perceptions of a Healthy Diet among Italian Consumers. Foods.

[43] Blom S.S., Gillebaart M., De Boer F., Van Der Laan N., De Ridder D.T. (2021). Under pressure: Nudging increases healthy food choice in a virtual reality supermarket, irrespective of system 1 reasoning. Appetite.

[44] Wanga X., Wangb X., Leic J., Chao M.C.-H. (2020). The clothes that make you eat healthy: The impact of clothes style on food choice. J. Bus. Res..

[45] Keegan E., Kemps E., Prichard I., Polivy J., Herman C.P., Tiggemann M. (2019). The effect of the spatial positioning of a healthy food cue on food choice from a pictorial-style menu. Eat. Behav..

[46] Klassen K.M., Douglass C.H., Brennan L., Truby H., Lim M.S.C. (2018). Social media use for nutrition outcomes in young adults: A mixed-methods systematic review. Int. J. Behav. Nutr. Phys. Act..

[47] Swindle T.M., Ward W.L., Whiteside-Mansell L. (2018). Facebook: The Use of Social Media to Engage Parents in a Preschool Obesity Prevention Curriculum. J. Nutr. Educ. Behav..

[48] Smith M.-K., Denali D.L. (2014). Social Media in Health Education, Promotion, and Communication: Reaching Rural Hispanic Populations along the USA/Mexico Border Region. J. Racial Ethn. Health Disparities.

[49] Mohammed W., Alanzi T., Alanezi F., Alhodaib H., AlShammari M. (2021). Usage of social media for health awareness purposes among health educators and students in Saudi Arabia. Inform. Med. Unlocked.

[50] Hu Y., Manikonda L., Kambhampati S. What We Instagram: A First Analysis of Instagram Photo Content and User Types. Proceedings of the 8th International Conference on Weblogs and Social Media, ICWSM 2014.

[51] Fleming-Milici F., Harris J.L. (2020). Adolescents’ engagement with unhealthy food and beverage brands on social media. Appetite.

[52] Simeone M., Scarpato D. (2020). Sustainable consumption: How does social media affect food choices?. J. Clean. Prod..

[53] Nelson A.M., Fleming R. (2019). Gender differences in diet and social media: An explorative study. Appetite.

[54] Hawks J.R., Madanat H., Walsh-Buhi E.R., Hartman S., Nara A., Strong D., Anderson C. (2020). Narrative review of social media as a research tool for diet and weight loss. Comput. Hum. Behav..

[55] Sutter C., Pham G.V., Yun J.T., Narang K., Sundaram H., Fiese B.H. (2021). Food parenting topics in social media posts: Development of a coding system, examination of frequency of food parenting concepts, and comparison across Reddit and Facebook. Appetite.

[56] Choudhary S., Nayak R., Kumari S., Choudhury H. (2019). Analysing acculturation to sustainable food consumption behaviour in the social media through the lens of information diffusion. Technol. Forecast. Soc. Chang..

[57] Steils N., Obaidalahe Z. (2020). “Social food”: Food literacy co-construction and distortion on social media. Food Policy.

[58] Zhou J., Liu F., Zhou H. (2018). Understanding health food messages on Twitter for health literacy promotion. Perspect. Public Health.

[59] Chau M.M., Burgermaster M., Mamykina L. (2018). The use of social media in nutrition interventions for adolescents and young adults—A systematic review. Int. J. Med. Inform..

[60] Schillinger D., Chittamuru D., Ramírez A.S. (2020). From “Infodemics” to Health Promotion: A Novel Framework for the Role of Social Media in Public Health. Am. J. Public Health.

[61] Otte E., Rousseau R. (2002). Social network analysis: A powerful strategy, also for the information sciences. J. Inf. Sci..

[62] Powell J., Hopkins M. (2015). A Librarian’s Guide to Graphs, Data and the Semantic Web.

[63] Pennacchiotti M., Popescu A.-M. Democrats, republicans and starbucks afficionados. Proceedings of the 17th ACM SIGKDD International Conference on Knowledge Discovery and Data Mining—KDD’11.

[64] Singh A., Halgamuge M.N., Moses B. (2019). An Analysis of Demographic and Behavior Trends Using Social Media: Facebook, Twitter, and Instagram. Social Network Analytics.

[65] Zeng D., Chen H., Lusch R., Li S.-H. (2010). Social Media Analytics and Intelligence. IEEE Intell. Syst..

[66] Jindal K., Aron R. (2021). A systematic study of sentiment analysis for social media data. Mater. Today Proc..

[67] Upadhyay N., Upadhyay S. (2017). RighttoBreathe why not? Social Media Analysis of the Local in the Capital City of India. Procedia Comput. Sci..

[68] Vidal L., Ares G., Machín L., Jaeger S.R. (2015). Using Twitter data for food-related consumer research: A case study on “what people say when tweeting about different eating situations”. Food Qual. Prefer..

[69] Chae B. (2015). (Kevin) Insights from hashtag #supplychain and Twitter Analytics: Considering Twitter and Twitter data for supply chain practice and research. Int. J. Prod. Econ..

[70] Culotta A. Estimating county health statistics with twitter. Proceedings of the SIGCHI Conference on Human Factors in Computing Systems.

[71] Widener M.J., Li W. (2014). Using geolocated Twitter data to monitor the prevalence of healthy and unhealthy food references across the US. Appl. Geogr..

[72] Xu C., Wong D.W., Yang C. (2013). Evaluating the “geographical awareness” of individuals: An exploratory analysis of twitter data. Cartogr. Geogr. Inf. Sci..

[73] Fayyad U., Piatetsky-Shapiro G., Smyth P. Knowledge Discovery and Data Mining: Towards a Unifying Framework. Proceedings of the Second International Conference on Knowledge Discovery and Data Mining.

[74] Chang H.-C., Iyer H. (2012). Trends in Twitter Hashtag Applications: Design Features for Value-Added Dimensions to Future Library Catalogues. Libr. Trends.

[75] Childers C.C., Lemon L.L., Hoy M.G. (2019). #Sponsored #Ad: Agency Perspective on Influencer Marketing Campaigns. J. Curr. Issues Res. Advert..

[76] De Veirman M., Cauberghe V., Hudders L. (2017). Marketing through Instagram Influencers: The Impact of Number of Followers and Product Divergence on Brand Attitude. Int. J. Advert..

[77] Pilař L., Stanislavská L.K., Pitrová J., Krejčí I., Tichá I., Chalupová M. (2019). Twitter Analysis of Global Communication in the Field of Sustainability. Sustainability.

[78] Pilar L., Moulis P., Pitrová J., Bouda P., Gresham G., Balcarová T., Rojík S. (2019). Education and Business as a key topics at the Instagram posts in the area of Gamification. J. Effic. Responsib. Educ. Sci..

[79] Stanislavská L.K., Pilař L., Margarisová K., Kvasnička R. (2020). Corporate Social Responsibility and Social Media: Comparison between Developing and Developed Countries. Sustainability.

[80] Twitter API v2: Early Access. https://developer.twitter.com/en/docs/twitter-api/early-access.

[81] Bastian M., Heymann S., Jacomy M. Gephi: An Open Source Software for Exploring and Manipulating Networks. Proceedings of the International AAAI Conference on Weblogs and Social Media.

[82] McCurdie T., Sanderson P., Aitken L.M. (2018). Applying social network analysis to the examination of interruptions in healthcare. Appl. Ergon..

[83] Newman M.E.J., Girvan M. (2004). Finding and evaluating community structure in networks. Phys. Rev. E.

[84] Knoke D., Yang S. (2008). Social Network Analysis.

[85] Blondel V.D., Guillaume J.-L., Lambiotte R., Lefebvre E. (2008). Fast unfolding of communities in large networks. J. Stat. Mech. Theory Exp..

[86] Smith A., Shneiderman B., Himelboim I. Mapping Twitter Topic Networks: From Polarized Crowds to Community Clusters. http://www.pewinternet.org/2014/02/20/mapping-twitter-topic-networks-from-polarized-crowds-to-community-clusters/.

[87] Bektas I., Kudubeş A.A., Ayar D., Bektas M. (2021). Predicting the healthy lifestyle behaviors of Turkish adolescents based on their health literacy and self-efficacy levels. J. Pediatr. Nurs..

[88] Karami A., Dahl A.A., Turner-McGrievy G., Kharrazi H., Shaw G. (2018). Characterizing diabetes, diet, exercise, and obesity comments on Twitter. Int. J. Inf. Manag..

[89] Ramage S., Farmer A., Eccles K.A., McCargar L. (2014). Healthy strategies for successful weight loss and weight maintenance: A systematic review. Appl. Physiol. Nutr. Metab..

[90] Cava E., Yeat N.C., Mittendorfer B. (2017). Preserving Healthy Muscle during Weight Loss. Adv. Nutr..

[91] Kelly Y., Zilanawala A., Booker C., Sacker A. (2018). Social Media Use and Adolescent Mental Health: Findings from the UK Millennium Cohort Study. EClinicalMedicine.

[92] Muñoz O.S. Marselis Ilonka Consumer Behavior as a Leverage Point in the Food System. https://www.metabolic.nl/publication/consumer-behavior-as-a-leverage-point-in-the-food-system/.

[93] Kerschke-Risch P. (2015). Vegan Diet: Motives, Approach and Duration. Ernahr. Umsch..

[94] Radnitz C., Beezhold B., DiMatteo J. (2015). Investigation of lifestyle choices of individuals following a vegan diet for health and ethical reasons. Appetite.

[95] Timko C.A., Hormes J.M., Chubski J. (2012). Will the real vegetarian please stand up? An investigation of dietary restraint and eating disorder symptoms in vegetarians versus non-vegetarians. Appetite.

[96] Oussalah A., Levy J., Berthezène C., Alpers D.H., Guéant J.-L. (2020). Health outcomes associated with vegetarian diets: An umbrella review of systematic reviews and meta-analyses. Clin. Nutr..

[97] Cramer H., Kessler C.S., Sundberg T., Leach M.J., Schumann D., Adams J., Lauche R. (2017). Characteristics of Americans Choosing Vegetarian and Vegan Diets for Health Reasons. J. Nutr. Educ. Behav..

[98] Laguna L., Fiszman S., Puerta P., Chaya C., Tárrega A. (2020). The impact of COVID-19 lockdown on food priorities. Results from a preliminary study using social media and an online survey with Spanish consumers. Food Qual. Prefer..

[99] Wolfson J.A., Ishikawa Y., Hosokawa C., Janisch K., Massa J., Eisenberg D.M. (2021). Gender differences in global estimates of cooking frequency prior to COVID-19. Appetite.

[100] Philippe K., Chabanet C., Issanchou S., Monnery-Patris S. (2021). Child eating behaviors, parental feeding practices and food shopping motivations during the COVID-19 lockdown in France: (How) did they change?. Appetite.

[101] Fonge Y.N., Jain V.D., Harrison C., Brooks M., Sciscione A.C. (2020). Examining the Relationship between Food Environment and Gestational Diabetes. Am. J. Obstet. Gynecol. MFM.

[102] Suarez J.J., Isakova T., Anderson C.A., Boulware L.E., Wolf M., Scialla J.J. (2015). Food Access, Chronic Kidney Disease, and Hypertension in the U.S. Am. J. Prev. Med..

[103] Korda H., Itani Z. (2011). Harnessing Social Media for Health Promotion and Behavior Change. Health Promot. Pr..

[104] Greenly L.W. (2004). A Doctor’s Guide to Diet Plans from A–Z. J. Chiropr. Med..

[105] Polli G.M., da Silveira F.M., Magnabosco F.M., dos Santos G.H.S., Stella P.D., Pinto P.R., Zibetti M.R., Apostolidis T. (2021). Representations of food among vegetarians in Brazil: A psychosocial approach. Int. J. Gastron. Food Sci..

[106] Saari U.A., Herstatt C., Tiwari R., Dedehayir O., Mäkinen S.J. (2021). The vegan trend and the microfoundations of institutional change: A commentary on food producers’ sustainable innovation journeys in Europe. Trends Food Sci. Technol..

[107] Lopez P.D., Cativo E.H., Atlas S.A., Rosendorff C. (2019). The Effect of Vegan Diets on Blood Pressure in Adults: A Meta-Analysis of Randomized Controlled Trials. Am. J. Med..

[108] Ploll U., Petritz H., Stern T. (2020). A social innovation perspective on dietary transitions: Diffusion of vegetarianism and veganism in Austria. Environ. Innov. Soc. Transit..

[109] Costa I., Gill P.R., Morda R., Ali L. (2019). “More than a diet”: A qualitative investigation of young vegan Women’s relationship to food. Appetite.

[110] Lemale J., Mas E., Jung C., Bellaiche M., Tounian P. (2019). Vegan diet in children and adolescents. Recommendations from the French-speaking Pediatric Hepatology, Gastroenterology and Nutrition Group (GFHGNP). Arch. Pédiatr..

[111] Lederer A.-K., Maul-Pavicic A., Hannibal L., Hettich M., Steinborn C., Gründemann C., Zimmermann-Klemd A.M., Müller A., Sehnert B., Salzer U. (2020). Vegan diet reduces neutrophils, monocytes and platelets related to branched-chain amino acids—A randomized, controlled trial. Clin. Nutr..

[112] Xiong Y., Cho M., Boatwright B. (2019). Hashtag activism and message frames among social movement organizations: Semantic network analysis and thematic analysis of Twitter during the MeToo movement. Public Relat. Rev..

[113] Tariq K., Serajuddin U. Should We Continue to Use the Term “Developing World”?. https://blogs.worldbank.org/opendata/should-we-continue-use-term-developing-world.

[114] North M., Kothe E., Klas A., Ling M. (2021). How to Define “Vegan”: An exploratory study of definition preferences among omnivores, vegetarians, and vegans. Food Qual. Prefer..

[115] Boursier V., Gioia F., Griffiths M.D. (2020). Selfie-engagement on social media: Pathological narcissism, positive expectation, and body objectification—Which is more influential?. Addict. Behav. Rep..

[116] Lou C., Yuan S. (2019). Influencer Marketing: How Message Value and Credibility Affect Consumer Trust of Branded Content on Social Media. J. Interact. Advert..

[117] Hansen T., Sørensen M.I., Eriksen M.-L.R. (2018). How the interplay between consumer motivations and values influences organic food identity and behavior. Food Policy.

[118] Janssen M., Busch C., Rödiger M., Hamm U. (2016). Motives of consumers following a vegan diet and their attitudes towards animal agriculture. Appetite.

[119] Bourcier E., Bowen D.J., Meischke H., Moinpour C. (2003). Evaluation of strategies used by family food preparers to influence healthy eating. Appetite.

[120] Peng M. (2019). The Growing Market of Organic Foods: Impact on the US and Global Economy. Safety and Practice for Organic Food.

[121] Seyfang G. (2007). Growing sustainable consumption communities. Int. J. Sociol. Soc. Policy.

